# Effect of Clonidine (an Antihypertensive Drug) Treatment on Oxidative Stress Markers in the Heart of Spontaneously Hypertensive Rats

**DOI:** 10.1155/2013/927214

**Published:** 2013-05-16

**Authors:** Nik Syamimi Nik Yusoff, Zulkarnain Mustapha, Chandran Govindasamy, K. N. S. Sirajudeen

**Affiliations:** Department of Chemical Pathology, School of Medical Sciences, Universiti Sains Malaysia, Health Campus, 16150 Kubang Kerian, Kelantan, Malaysia

## Abstract

Hypertension is a risk factor for several cardiovascular diseases and oxidative stress suggested to be involved in the pathophysiology. Antihypertensive drug Clonidine action in ameliorating oxidative stress was not well studied. Therefore, this study investigate the effect of Clonidine on oxidative stress markers and nitric oxide (NO) in SHR and nitric oxide synthase inhibitor, N-nitro-L-arginine methyl ester (L-NAME) administered SHR. Male rats were divided into four groups [SHR, SHR+Clonidine (SHR-C), SHR+L-NAME, SHR+Clonidine+L-NAME(SHRC+L-NAME)]. Rats (SHRC) were administered with Clonidine (0.5 mg kg^−1^ day^−1^) from 4 weeks to 28 weeks in drinking water and L-NAME (25 mg kg^−1^ day^−1^) from 16 weeks to 28 weeks to SHRC+L-NAME. Systolic blood pressure (SBP) was measured. At the end of 28 weeks, all rats were sacrificed and in their heart homogenate, oxidative stress parameters and NO was assessed. Clonidine treatment significantly enhanced the total antioxidant status (TAS) (*P* < 0.001) and reduced the thibarbituric acid reactive substances (TBARS) (*P* < 0.001) and protein carbonyl content (PCO) (*P* < 0.05). These data suggest that oxidative stress is involved in the hypertensive organ damage and Clonidine not only lowers the SBP but also ameliorated the oxidative stress in the heart of SHR and SHR+L-NAME.

## 1. Introduction

Globally, near one billion people have hypertension; of these, two-thirds are in developing countries, killing nearly 8 million people worldwide every year and nearly 1.5 million people each year in South-East Asia region approximately one-third of the adult population in this region has high blood pressure [1]. The Sixth Reports of the Joint National Committee on Prevention, Detection, Evaluation, and Treatment of High Blood Pressure (JNC VI) define hypertension or high blood pressure to be present if it is persistently at or above 140/90 mmHg.

Hypertension is a risk factor for several cardiovascular diseases such as atherosclerosis or myocardial infarction [2], kidney failure [3], stroke, and death [4]. 

Growing evidence indicated that oxidative stress plays an important role in the pathophysiology of hypertension [5]. Hypertension is associated with imbalance of oxidant antioxidant status which causes alteration in lipid peroxidation [6] superoxide dismutase and glutathione peroxidase [7] and higher production in hydrogen peroxide [8]. 

Nitric oxide (NO) (endothelium-derived relaxing factor) is synthesised in biological system by nitric oxide synthase (NOS) and has a strong vasodilatory effect [9]. NOS inhibitor such as N-nitro-L-arginine methyl ester (L-NAME) has a causal role in oxidative stress [10–12] as well as promotes persistent hypertension and progressive cardiovascular damage [13]. Zhou and Frohlich [13] developed and established the L-NAME/SHR model that mimics the pathophysiological alterations associated with naturally-occurring progressive impairment of cardiac and renal functions and structure. They have reviewed series of studies designed using the L-NAME/SHR to investigate the effect of antihypertensive agents on prevention, development, progression, and even reversal of hypertensive nephrosclerosis. Each report documented the pathophysiological actions by the intervention of an antihypertensive agent either concomitantly with or subsequently following L-NAME. However, the studies were focusing on pathophysiological effect of calcium antagonists, angiotensine converting enzyme inhibitors and aldosterone antagonist. Therefore, using the L-NAME/SHR model, we would like to determine the effects of Clonidine, an a-adrenoceptor agonists, another class of antihypertensive drug on oxidative stress markers [thiobarbituric acid reactive substances (TBARS), protein carbonyl (PCO), total antioxidant status (TAS)] and nitric oxide level in the heart of SHR and SHR+L-NAME. 

## 2. Methods

This study was approved by the Animal Ethics and Welfare Committee of Universiti Sains Malaysia. Male SHR aged 4 weeks were obtained from the Animal Research and Service Centre (ARASC), Health Campus Universiti Sains Malaysia, and housed in individual cages in standard environment (25–27°C) room temperature under 12 hours light and 12 hours dark cycle (lights on 0700–1900 hours). The animals were fed with commercial rat food pellet and Clonidine (Sigma, USA) was given through drinking water. Rats were divided into 4 groups: (1) SHR (untreated), (2) SHR treated with Clonidine (0.5 mg kg^−1^ day^−1^; 4–28 weeks) (SHRC), (3) SHR administered L-NAME (25 mg kg^−1^ day^−1^) (untreated)(SHR/L-NAME), and (4) SHR administered L-NAME (25 mg kg^−1^ day^−1^) treated with Clonidine (0.5 mg kg^−1^ day^−1^; 4–28 weeks) (SHRC/L-NAME) of which each group consists of 6 animals (*n* = 6). Chronic administration of L-NAME started in rats aged 16 weeks until 28 weeks in group 3 and group 4. The normotensive Wistar-Kyoto (WKY) rats were divided and treated with clonidine in a similar manner with SHR groups.

Systolic blood pressure (SBP) was taken during the experimental period for every two weeks using the tail plethysmography blood pressure analyzer (IITC Life Science, USA). Rats were weighed and sacrificed at the end of 28th weeks. The heart was collected and homogenized (Glas-Col, USA) in 0.05 M sodium phosphate buffer (pH 7.4). Supernatant of heart homogenate was stored at −70°C until use for biochemical analysis.

Lipid peroxidation was determined as thiobarbituric acid reactive substances (TBARS) according to the method of Chatterjee et al. [14]. MDA, an end product of fatty acid peroxidation, react with TBA to form coloured complex which has maximum absorbance at 532 nm. 1,1,3,3-Tetraethoxypropane (TEP); a form of MDA was used as standard in this assay. 0.1% of heart homogenate or MDA standard (2 *μ*M, 4 *μ*M, 6 *μ*M, and 8 *μ*M) were pipette into each test tubes. The test tubes were vortexed and then kept in boiling water bath at 95°C for 60 minutes. After cooling, the tubes were centrifuged at 3000 ×g for 10 minutes. One mL of each supernatant was transferred to semimicrocuvette and absorbance was read at 532 nm on a spectrophotometer.

Protein carbonyl (PCO) levels were determined using Protein Carbonyl Assay Kit (Cayman, USA) according to the method of Rohrbach et al. [15]. DNHP react with protein carbonyl, forming a Schiff base to produce corresponding hydrazone. The amount of protein-hydrazone produced was quantified spectrophotometrically at an absorbance between 360 and 385 nm. 

Total antioxidant status (TAS) was assessed according to the method of Koracevic et al. [16]. It was based on the principle that a standardized solution of Fe-EDTA complex reacted with hydrogen peroxide by a Fenton-type reaction, leading to the formation of hydroxyl radicals. These reactive oxygen species degraded benzoate, resulting in the release of TBARS. Antioxidants from the added sample of heart homogenate caused suppression of the production of TBARS that was proportional to their concentration. This reaction was measured spectrophotometrically at 532 nm and the inhibition of colour development was defined as the TAS.

Nitric Oxide (NO) was determined using Nitrate/Nitrite Colorimetric Assay Kit according to the method Yui et al. [17]. Nitrate reductase utilizes NADPH in the enzymatic reduction of nitrate to nitrite. Nitrite produced reacts with Griess Reagent 1 followed by Griess Reagent 2 to produce Azo product. The concentration of the Azo product in the sample was obtained by measuring the absorbance at 540 nm. 

## 3. Statistical Analysis

Data were analyzed using One-Way ANOVA with post hoc Tukey test. Data were analyzed using Statistical Package for the Social Science (SPSS) software version 20. Significant level was set at (*P* < 0.05). Data are expressed as mean and standard error mean (mean ± SEM) for six animals in each group.

## 4. Results

### 4.1. SBP, Oxidative Stress Parameters, and NO of Clonidine Treated and Untreated SHR and SHR+L-NAME

**Figure 1:**
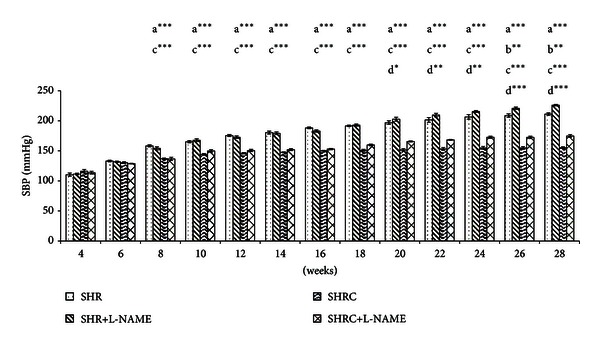
SBP of Clonidine treated and untreated SHR administered with L-NAME. a***  *P* < 0.001 SHR compared to SHRC, b**  *P* < 0.01 SHR+L-NAME compared to SHR, c***  *P* < 0.001 SHRC+L-NAME compared to SHR+L-NAME and d*  *P* < 0.05, d**  *P* < 0.01, and d***  *P* < 0.001 SHRC+L-NAME compared to SHRC.

The SBP of Clonidine treated and untreated SHR and SHR+L-NAME were presented in Figure 1. SBP of SHR treated with Clonidine (SHRC) were significantly lower from age of 8 weeks until 28 weeks when compared to untreated SHR (*P* < 0.001, a***). L-NAME was administered to rats at age of 16 weeks. Therefore, SHR+L-NAME treated with Clonidine (SHRC+L-NAME) showed significant increase compared to untreated SHR+L-NAME (c***, *P* < 0.001), before L-NAME was administered as these still represent the SHRC compared to SHR. After administration of L-NAME, SHR+L-NAME showed significant increase in SBP in weeks 26 (*P* < 0.01, b**) and in weeks 28 (*P* < 0.001, b***) compared to SHR. There was also significant decrease of SBP in Clonidine treated SHR administered L-NAME (SHRC+L-NAME) when compared to untreated SHR+L-NAME from weeks 20 until weeks 28 (week 20: *P* < 0.05, d*, week 22 and 24: *P* < 0.01, d**, and week 26 & 28: *P* < 0.001, d***).

### 4.2. TAS

**Figure 2:**
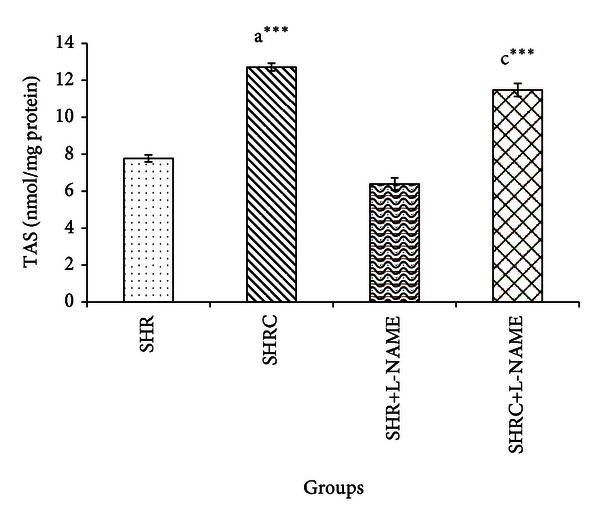
TAS levels in Clonidine treated and untreated SHR administered with L-NAME. a***  *P* < 0.001 SHR compared to SHRC, c***  *P* < 0.001 SHRC+L-NAME compared to SHR+L-NAME.

Figure 2 represents the level of TAS in Clonidine treated and untreated SHR and SHR+L-NAME. The level of TAS was significantly increased with Clonidine treatment in SHR (*P* < 0.001, a***). However, no significant difference in TAS level in L-NAME administered SHR when compared to SHR. Significant increase was evident in TAS level in Clonidine treated SHR administered with L-NAME (SHRC+L-NAME) when compared to untreated SHR administered L-NAME (SHR+L-NAME) (*P* < 0.001, c***). No significant difference was observed in SHRC+L-NAME when compared to Clonidine treated SHR without L-NAME (SHRC).

### 4.3. TBARS

**Figure 3:**
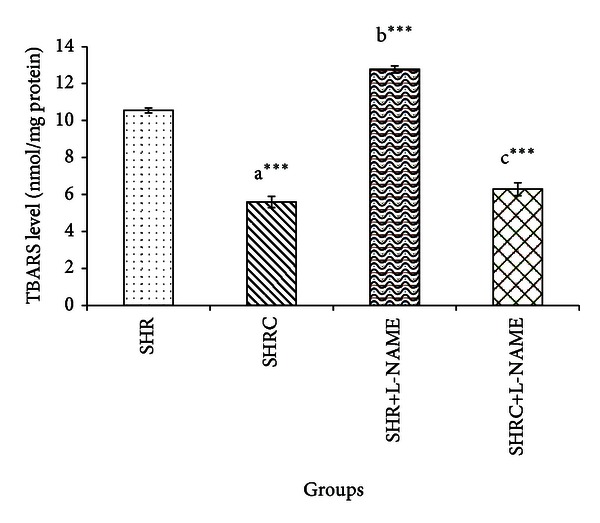
TBARS levels in Clonidine treated and untreated SHR administered with L-NAME. a***  *P* < 0.001 SHR compared to SHRC, b***  *P* < 0.001 SHR+L-NAME compared to SHR, and c***  *P* < 0.001 SHRC+L-NAME compared to SHR+L-NAME.

The TBARS levels of Clonidine treated and untreated SHR and SHR+L-NAME were provided in Figure 3. TBARS level in Clonidine treated SHR was significantly lower compared to untreated SHR (*P* < 0.001, a***). The levels of TBARS in SHR administered with L-NAME (SHR+L-NAME) were significantly higher when compared to SHR (*P* < 0.001, b***). Significant decrease was evident in TBARS level in Clonidine treated SHR administered with L-NAME (SHRC+L-NAME) when compared to untreated SHR administered L-NAME (SHR+L-NAME) (*P* < 0.001, c***). No significant difference was observed in SHRC+L-NAME when compared to Clonidine treated SHR without L-NAME (SHRC).

### 4.4. PCO

**Figure 4:**
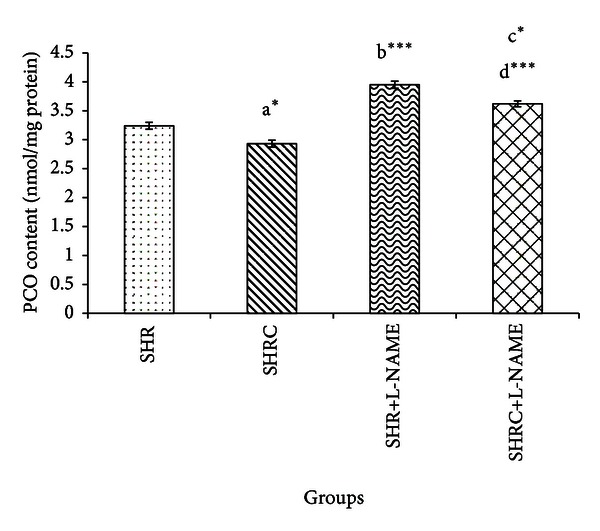
PCO levels in Clonidine treated and untreated SHR administered with L-NAME. a*  *P* < 0.05 SHR compared to SHRC, b***  *P* < 0.001 SHR+L-NAME compared to SHR, c*  *P* < 0.05 SHRC+L-NAME compared to SHR+L-NAME, and d***  *P* < 0.001 SHRC+L-NAME compared to SHRC.

Figure 4 represents the PCO level of Clonidine treated and untreated SHR and SHR+L-NAME. PCO level in Clonidine treated SHR was significantly lower compared to untreated SHR (*P* < 0.05, a*). Significant increase was observed in PCO levels of SHR administered L-NAME (SHR+L-NAME) when compared to SHR (*P* < 0.001, b***). Significant decrease was evident in PCO level in Clonidine treated SHR administered with L-NAME (SHRC+L-NAME) when compared to untreated SHR administered L-NAME (SHR+L-NAME) (*P* < 0.05, c*). Significant increase also was observed in SHRC+L-NAME when compared to Clonidine treated SHR without L-NAME (SHRC) (*P* < 0.001, d***).

### 4.5. NO

**Figure 5:**
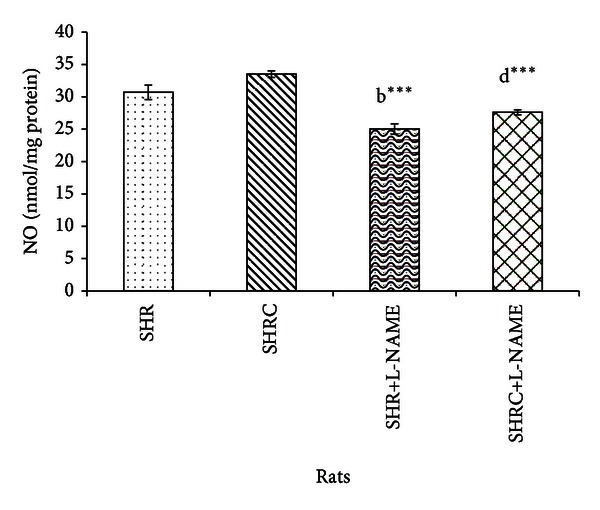
NO levels in Clonidine treated and untreated SHR administered with L-NAME. b***  *P* < 0.001 SHR+L-NAME compared to SHR and d***  *P* < 0.001 SHRC+L-NAME compared to SHRC.

The NO levels of Clonidine treated and untreated SHR and SHR+L-NAME were given in Figure 5. NO level in Clonidine treated SHR and SHR+L-NAME was increased but not statistically significant compared to untreated SHR. Significant decrease was observed in NO levels of SHR administered L-NAME (SHR+L-NAME) when compared to SHR (*P* < 0.001, b***). No significant difference was evident in NO level in Clonidine treated SHR administered with L-NAME (SHRC+L-NAME) when compared to untreated SHR administered L-NAME (SHR+L-NAME). Significant decrease also was observed in SHRC+L-NAME when compared to Clonidine treated SHR without L-NAME (SHRC) (*P* < 0.001, d***).

### 4.6. SBP, Oxidative Stress Parameters, and NO of Clonidine Treated and Untreated WKY and WKY+L-NAME

WKY rats showed normal SBP. As expected, L-NAME administration in WKY significantly increases the SBP at 18 weeks (*P* < 0.05) and 20 weeks onward (*P* < 0.001) when compared to WKY. Clonidine treatment significantly reduced the SBP in WKY+L-NAME when compared to WKY+L-NAME untreated (data not shown). 

Table 1 showed the oxidative stress parameters and NO of normotensive WKY rats. Significant increase (b***, *P* < 0.001) was evident in TBARS and PCO levels of WKY+L-NAME when compared to WKY. However, no significant difference was evident in TAS levels of WKY+L-NAME when compared to WKY. Clonidine treatment significantly elevated the TAS level in WKY when compared to untreated WKY (a***, *P* < 0.001), but no significant difference was seen in PCO levels. TBARS level showed significant increase in Clonidine treated WKY (a*, *P* < 0.05) and WKY+L-NAME (c*, *P* < 0.05) when compared to their matched untreated groups. Significant decrease (b*** and d***, *P* < 0.001) in NO level was evident in L-NAME administered groups. However, no significant difference was evident in NO level in Clonidine treated WKY and WKY+L-NAME when compared to their matched untreated groups. 

## 5. Discussions

The results of this study showed that SBP was significantly elevated in SHR as demonstrated in previous studies [18, 19]. Administration of L-NAME at 16 weeks initially did not elevate SBP in SHR. However, at weeks 26 and 28, SBP was elevated in SHR+L-NAME. Gerová et al. [20] showed that the use of L-NAME as NOS inhibitor alters the regulation of blood pressure and is accompanied by development of cardiovascular disorders. It has been shown that chronic reduction of NO synthesis resulted in hypertension [20, 21] and reduced endothelial vasorelaxation [22–24] and myocardial hypertrophy [25]. The results of this study demonstrated that Clonidine can attenuate development of high blood pressure and end-organ damage in SHR and SHR+L-NAME with significant endothelial dysfunction produced by inhibition of NO synthesis. Since 1960, Clonidine is believed to lower blood pressure through several modes of action such as stimulating a-adrenoceptors in the cardiovascular centers with a reduction of sympathetic nerve activity and noradrenaline release [26, 27] producing suppressant effect on adrenal medullary function, and decreasing adrenal sympathetic nerve activity [28–30], and activation of nonadrenergic depressor pathways, and activation of baroreflex [31, 32]. 

Clonidine treatment also delayed the onset and attenuated the severity of hypertension produced by L-NAME inhibition. Rizzoni et al. reported that SHR are normotensive at 4 weeks of age and develop hypertension, cardiac hypertrophy and vascular dysfunction by 8–12 weeks [33]. 

The increase in the systolic blood pressure in SHR was accompanied by changes in the marker of oxidative stress, such as lower heart total antioxidant status, higher thiobarbituric acid reactive substance, and elevation of protein carbonyl content. This implies, as previously demonstrated, an increase in the production of reactive oxygen species, which in turn produce a higher systemic and heart oxidative stress in SHR [5]. Alteration in oxidant-antioxidant balance in SHR demonstrated in this study may contribute to the generation and/or maintenance of hypertension via promoting vascular smooth muscle cell proliferation and hypertrophy as well as collagen deposition, leading to thickening of vascular media and narrowing of the vascular lumen as reviewed by Grossman [34]. In addition, increase oxidative stress may damage the endothelium, impair endothelium-dependent vascular relaxation, and increase vascular contractile activity [35]. 

The present study demonstrates that nitric oxide synthase inhibition by L-NAME reduced the nitric oxide level in SHR. By contrast, administration of L-NAME was not affecting the TAS. L-NAME inhibits the production of vasodilator nitric oxide. 

Regarding Clonidine treatment, data from the study herein revealed that Clonidine treatment elevated total antioxidant status in SHR. To further evaluate the effect of Clonidine against hypertension-associated oxidative stress, the lipid peroxidation product (TBARS) and protein oxidation product (PCO) in heart were examined. Clonidine treatment decreases the level of lipid peroxidation product and protein oxidation product in heart of SHR and SHR administered L-NAME in comparison to untreated groups. 

The protective effect of clonidine may have multiple components in its action. Clonidine, an *α*2-adrenergic receptor agonist, is thought to decrease blood pressure by causing a reduced sympathetic nerve firing rate with the locus of its action within the CNS [36]. The same report also demonstrated that Clonidine treatment results in hypotensive in SHR through action of sympathetic vasoconstrictor fibers. *α*2-Adrenoceptor agonists, such as clonidine, attenuate hypoxia-induced damage to brain and retinal neurones by a mechanism of action which likely involves stimulation of a-adrenoceptors. When compared to Clonidine administered WKY and WKY+L-NAME (Table 1), Clonidine administered SHR and SHR+L-NAME showed a significant reduction in oxidative stress parameters which clearly indicate that Clonidine is reducing oxidative stress in SHR and SHR+L-NAME. 

From this study, therefore, it concluded that some evident was found to support the idea that, in addition to the hypotensive effect of Clonidine, this antihypertensive drug enhance the level of antioxidant status and ameliorate the oxidative stress which might reduce the hypertension induced heart damage in SHR and SHR+L-NAME. A better understanding of the complexity of oxidative stress and hypertension, role of nitric oxide, and development of new class of antihypertensive drug which ameliorates oxidative stress will likely be the avenues for future research to prevent hypertension and hypertension induced cardiovascular complications. 

## Figures and Tables

**Table 1:** Effect of Clonidine treatment on TBARS, PCO, TAS, and NO level of Clonidine treated and untreated WKY and WKY administered L-NAME.

Parameters	Groups			
	WKY	WKY+L-NAME	WKYC	WKYC+L-NAME
TAS	7.64 ± 0.17	6.91 ± 0.05	10.54 ± 0.93 a***	8.53 ± 0.18
TBARS	5.59 ± 0.32	7.49 ± 0.20 b***	7.80 ± 0.49 a*	8.98 ± 0.25 c*
PCO	2.69 ± 0.07	3.12 ± 0.05 b***	2.94 ± 0.18	3.11 ± 0.19 c*
NO	37.65 ± 0.56	27.92 ± 0.79 b***	39.25 ± 0.65	30.71 ± 0.69 d***

Values are expressed as mean ± S.E.M. (*n* = 6 per group).

WKY: WKY no treatment, WKY+L-NAME: WKY no treatment+L-NAME, WKYC: WKY+Clonidine, and WKYC+L-NAME: WKY+Clonidine+L-NAME.

a*  *P* < 0.05, a***  *P* < 0.001 WKY compared to WKYC, b***  *P* < 0.001 WKY+L-NAME compared to WKY, c*  *P* < 0.05 WKYC+L-NAME compared to WKY+L-NAME, and d***  *P* < 0.001 WKYC+L-NAME compared to WKYC.
